# Microbials for the production of monoclonal antibodies and antibody fragments

**DOI:** 10.1016/j.tibtech.2013.10.002

**Published:** 2014-01

**Authors:** Oliver Spadiut, Simona Capone, Florian Krainer, Anton Glieder, Christoph Herwig

**Affiliations:** 1 Vienna University of Technology, Institute of Chemical Engineering, Research Area Biochemical Engineering, Gumpendorfer Strasse 1a, A-1060 Vienna, Austria; 2 Graz University of Technology, Institute of Molecular Biotechnology, Graz, Austria; 3 Austrian Centre of Industrial Biotechnology (ACIB GmbH), Graz, Austria

**Keywords:** monoclonal antibody, antibody fragment, mammalian cell, microbial organism, recombinant protein production

## Abstract

Monoclonal antibodies (mAbs) and antibody fragments represent the most important biopharmaceutical products today. Because full length antibodies are glycosylated, mammalian cells, which allow human-like N-glycosylation, are currently used for their production. However, mammalian cells have several drawbacks when it comes to bioprocessing and scale-up, resulting in long processing times and elevated costs. By contrast, antibody fragments, that are not glycosylated but still exhibit antigen binding properties, can be produced in microbial organisms, which are easy to manipulate and cultivate. In this review, we summarize recent advances in the expression systems, strain engineering, and production processes for the three main microbials used in antibody and antibody fragment production, namely *Saccharomyces cerevisiae*, *Pichia pastoris*, and *Escherichia coli*.

## Highlights

• Glycosylated full length antibodies are currently produced in mammalian cells.
• Antibody fragments can be produced in microbial organisms.
• Strain engineering allows production of full length antibodies in microbials.
• Microbials provide several advantages over mammalian cells.

## Introduction

Over the past three decades, the biopharmaceutical market has become a significant component of the global pharmaceutical market accounting for around 40% of its sales. The use of organisms as biopharmaceutical production factories offers several advantages over chemical synthesis. Microorganisms can produce high molecular weight compounds such as proteins [1] and carry out highly enantio- and regio-selective reactions by their native enzymatic machinery – these reactions are hard to achieve by chemical synthesis. The use of microorganisms also enables repeated implementation of immobilized enzymes or cells resulting in the reduction of the overall production costs [2]. Finally, processes employing microorganisms do not generate organic and inorganic pollutants, such as mercury and toluene [3].

**Figure 1:**
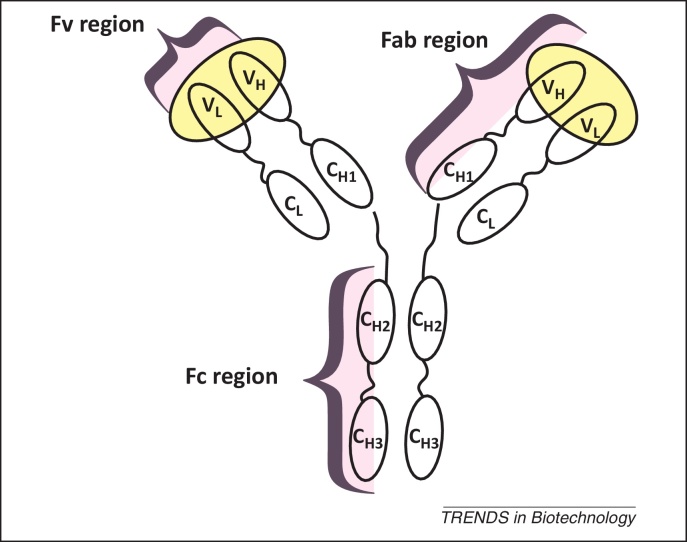
Schematic view of a full length antibody (the antigen binding sites are highlighted in yellow).

The biopharmaceutical market originated in the late 1970s with the establishment of recombinant DNA techniques. The industrial interest materialized almost immediately and in 1982 the US Food and Drug Administration (FDA) approved the commercialization of humulin, the human insulin analog, recombinantly produced in the bacterium *E. coli* [4]. For a while the FDA only allowed the transformation of bacteria and the expression of small, non-glycosylated proteins, like insulin, due to concern about introducing new toxicities such as contaminating bacterial substances, which raise immunogenic reactions in patients. However, with the development of selectable resistance markers, like antibiotic resistance markers, and the possibility of production in eukaryotic organisms, the FDA began showing increasing flexibility towards biotechnological innovation, leading to a continually increasing number of approved new biological entities (NBEs). In 2012, the biopharmaceutical market turnover was estimated at around 100–120 billion US dollars per year [5], with more than 200 biopharmaceutical proteins already on the market [6], and is expected to reach 170 billion US dollars in 2014. This exceptionally high market turnover is largely derived from the marketing of mAbs and antibody fragments that currently represent the fastest growing class of approved biopharmaceutical products. In fact, production of full length mAbs (Figure 1) is the most important biopharmaceutical venture to date, with several therapeutic products reaching blockbuster status (e.g., Avastin, Herceptin, Remicade, Rituxan, Humira, and Erbitux).

More recently, interest has grown in the production of antibody fragments that can be used not only in therapeutic applications but also in immunodetection, purification, and bioseparation applications [7]. Antibody fragments still exhibit antigen binding properties and can be produced in microbials, which are easy to manipulate and cultivate. In this review, we summarize recent advances in the expression system, strain engineering, and production process for the three main microbials for antibody fragment production, namely *S. cerevisiae*, *P. pastoris*, and *E. coli*, and highlight ongoing research that may allow full length antibody production in these organisms in the future.

## mAbs and antibody fragments: an overview

A full length mAb consists of the constant Fc (crystallizable fragment) domain and an antigen binding domain, comprising the Fv (variable fragment) and the Fab region (antibody binding fragment; Figure 1). Native full length mAbs are glycosylated during their synthesis. Although the glycosylated Fc domain does not directly interact with antigens, it stabilizes the antibody and is important for antibody-dependent, cell-mediated cytotoxicity. Moreover, glycosylation strongly impacts the clearance rate of the recombinant mAb from the body, and incompatible glycoforms can cause severe immunogenic effects in patients. Thus, much current work is focused on optimizing and controlling glycosylation events in mammalian cells [8], which at this time are the most often used cell type for the production of mAbs (Box 1).

Box 1 **Production of mAbs in mammalian cells: advantages and drawbacks**

Mammalian cells are used most often for production of mAbs due to their ability to perform post-translational modifications (PTM), especially human-like N-glycosylation. Their use simplifies subsequent medical applications by eliminating the risk of an immunogenic response in patients due to incompatible N-glycans on the protein. Chinese Hamster Ovary (CHO) cell lines are used most frequently to generate full length mAbs with human-like Fc N-glycosylation and production titers of around 10 g/l [8]. However, the use of mammalian cells for heterologous protein expression holds several drawbacks such as low product yield and growth rate, risk of viral contamination, and requirement for serum. Despite the introduction of serum-free (SF) chemically defined media (CDM) encountering regulatory requirements [56], the addition of chemically undefined hydrolysates is still necessary to support cell growth. This, however, highly contradicts QbD guidelines demanding defined growth media [57]. Furthermore, the current standard production process is cumbersome and time-consuming. Cell transfection leads to high clone heterogeneity, necessitating repeated screening procedures at increasing drug concentrations for the isolation of a positive, highly productive clone [8]. Clone evaluation and culture condition optimization is then performed in shake flasks and lab-scale bioreactors before production processes can be set up. However, scale-up is also very challenging. The catabolism of the main carbon sources, glucose and glutamine, leads to formation of the inhibiting metabolites lactate and ammonium, respectively; hence batch and fed-batch operation modes, both representing closed cultivation systems, are only possible for a restricted timeframe. Because the metabolism of mammalian cells is highly sensitive and responsive to changing culture conditions, bioprocesses are hard to model – in fact only unstructured models are possible – and to control, which again contradicts QbD guidelines [57]. Consequently, chemostat cultivations, which describe open cultivation systems where substrate is constantly fed and cultivation broth is continuously removed, are generally employed to avoid metabolite inhibition. To avoid a critical wash out of mammalian cells, perfusion systems that provide cell retention by employing membranes are mainly used. However, operating a continuous culture with a perfusion system requires more devices and control systems than a batch or fed-batch system and also bears the elevated risk of contamination. Another drawback associated with scaling-up mammalian cell cultures is their sensitivity to shear stress, creating further challenges to efficient aeration in large vessels. Thus, although mammalian cells can produce mAbs with compatible PTMs, several drawbacks in bioprocessing are yet to be overcome.

Nevertheless, a full length antibody with a glycosylated Fc domain is not necessary for antigen recognition. In fact, both the Fv and the Fab region alone (Figure 1) exhibit antigen binding properties. Furthermore, antibody fragments show increased tissue penetration and a lower retention time in non-target tissues compared to mAbs [9]. Although the lack of the stabilizing Fc domain causes reduced stability [10], the absence of glycosylation on both the Fv and the Fab regions allows their production to be less complex and enables easier engineering and cultivation of microbial host organisms such as bacteria and yeasts.

## Microbial expression hosts for mAbs and antibody fragments

### The yeast S. cerevisiae

*S. cerevisiae* was the first yeast employed in the production of recombinant proteins, and several biopharmaceuticals produced in this yeast have since been successfully marketed [11]. There are several intrinsic characteristics, like the stability of the expression system and the ease of cultivation, as well as advances in host engineering, that make *S. cerevisiae* an attractive host for the production of mAbs and antibody fragments. In fact, the production of Llama heavy chain antibody fragments (Hvv) in *S. cerevisiae* already represents a well-established industrial process, ensuring production titers up to hundreds of mg/l [12].

#### Expression system

*S. cerevisiae* is easy to transform either chemically or by electroporation. There are three main types of shuttle vectors in use: (i) yeast episomal plasmids (Yep), which contain the 2 μ origin of replication, allowing gene expression without genomic integration at high copy numbers; (ii) yeast centromeric plasmids (Ycp), which contain an autonomously replicating sequence and replicate with single or very low gene copy number; and (iii) yeast integrative plasmids (Yip), which lack the yeast origin of replication and are integrated into the host genome [13]. Although genomic integration of the target gene leads to a reduced expression level, it is highly desirable in terms of process quality and stability [14]. To overcome the disadvantage of low expression, targeted integration of the heterologous gene at the highly transcribed ribosomal DNA locus was developed recently [15]. In addition, commonly used promoters derived from the native glycolytic pathway, such as the promoters for glyceraldehyde-3-phosphate dehydrogenase (GAP), alcohol dehydrogenase1 (ADH1), phosphoglycerate kinase (PGK), and phosphoglycerate kinase (PGK1), allow high transcription levels [16]. Finally, new cloning strategies introduced recently allow the concomitant expression of two or more genes located on specially designed self-replicating plasmids [17], which also addresses the issue of low expression levels of heterologous genes caused by genomic integration.

#### Strain engineering

Despite continuing advances in genetic manipulation, efficient production of mAbs and antibody fragments in *S. cerevisiae* can still be impaired by endoplasmic reticulum (ER) misfolding and inefficient trafficking. Although Hvv can be produced successfully in sufficient amounts [12], the expression of the significantly smaller single chain Fv (scFv) region (Figure 1) leads to intracellular accumulation of misfolded proteins in the ER or in vacuolar-like organelles. A possible explanation for this is the higher hydrophobicity of the variable light and heavy chains of scFv compared to Hvv [18]. However, additional overexpression of chaperones and foldases can correct protein folding and allow subsequent scFv secretion [19].

Several strategies have been developed to increase the overall secretory capacity and productivity of *S. cerevisiae*. These approaches include engineering intracellular protein trafficking by over-expression of soluble N-ethylmaleimide-sensitive factor (NFS) attachment protein receptor proteins (SNAREs) [20], reduction of proteolytic degradation by multiple protease gene deletions [21], and engineering of the heat shock response (HSR) pathway by overexpressing the heat shock transcription factor (Hsf) [22]. Although these engineered strains have not yet been used for the production of mAbs and antibody fragments, they demonstrate the ongoing, intensive strain engineering work that is being done with *S. cerevisiae*.

#### Production process

Production of antibody fragments in *S. cerevisiae* is generally done in glucose-limited fed-batch cultivations [12]. Yeast shows a mixed oxidative/fermentative metabolism, which can result in the undesired production of toxic metabolites. Fermentative mode shift is triggered by oxygen depletion or by elevated carbon source concentration. Limiting glucose is therefore a valid strategy for preventing fermentation during cultivation processes with this yeast. Recently, a fully aerobically engineered strain, in which glucose uptake was reduced, was developed, allowing a full aerobic respiration even at elevated glucose concentrations [23].

As this discussion indicates, there are ongoing efforts to optimize the yeast *S. cerevisiae* for the production of mAbs and antibody fragments. Because antibody fragments are not glycosylated, they can be produced successfully in this yeast and are not affected by hypermannosylation, which characterizes *S. cerevisiae* [24]. Furthermore, current studies are investigating the possibility of humanizing the glycosylation machinery in *S. cerevisiae* [25], in an attempt to engineer this yeast for the production of full length mAbs.

### The yeast P. pastoris

As an alternative to *S. cerevisiae*, the methylotrophic yeast *P. pastoris*, which is closely related to *S. cerevisiae*, can be used for the production of mAbs and antibody fragments as it also holds a generally recognized as safe (GRAS) status [26].

#### Expression system

Similar to the process in *S. cerevisiae*, the target gene is integrated into the genome of *P. pastoris* to guarantee reproducibility and stability of the expression system. However, a major obstacle in *P. pastoris* is the substantial degree of non-homologous recombination. One solution to this challenge is the use of a recently developed *P. pastoris* strain with an inactivated non-homologous end joining pathway [27].

*P. pastoris* can use methanol as a sole carbon source, as it is a crucial part of its metabolism (e.g., [28]). However, instead of the traditional hard-to-control alcohol oxidase promoter system typically used for *P. pastoris*, alternative adjustable promoters are currently under investigation [29]. Furthermore, the generation of artificial and semi-artificial, tunable promoter variants are the subject of recent synthetic biology approaches [30].

#### Strain engineering

The genome sequences of the wild type strains NRRL Y-1603 (identical to DSMZ 70382 or CBS704) [7], NRRL Y-11430 (identical to ATCC 7673 or CBS7435), and GS115 are available online 31, 32 and a genome-scale metabolic model of *P. pastoris* was published recently [33], allowing straight-forward strain engineering approaches. For example, co-overexpression of helper proteins, such as the protein disulfide isomerase or the transcription factor of the unfolded protein response Hac1 [34], as well as inactivation of endogenous proteases (e.g., [35]) enhances the production and secretion of recombinant proteins. Engineering the protein trafficking pathway represents another successful approach to improve secretion [36]. In addition, intensive glycoengineering work is ongoing to humanize the glycosylation events in *P. pastoris* and allow production of full length mAbs in this yeast (Box 2).

Box 2 **Glycoengineering of Pichia pastoris allows mAb production**

*P. pastoris* can be used for the production of both antibody fragments and mAbs (e.g., [58]). For mAbs, the correct human-type glycosylation is not only essential for proper folding and biological activity, but also for targeting and stability in circulation. *P. pastoris* lacks the Golgi-resident α-1,3-mannosyltransferase, but harbors four additional β-mannosyltransferases instead 59, 60. The absence of terminal α-1,3-mannoses on *P. pastoris*-derived glycoproteins is of importance because this glycan structure causes high antigenicity in humans [61]. Thus, the humanization of the N-glycosylation pathway in *P. pastoris* has been an important goal. The Outer CHain elongation 1 gene (*OCH1*) coding for an α-1,6-mannosyltransferase was knocked out [62], and an α-1,2-mannosidase, β-N-acetylglucosaminyltransferase I (GnTI) and an UDP-GlcNAc transporter were introduced [63]. The *Kluyveromyces lactis* UDP-GlcNAc transporter, mouse α-1,2-mannosidase IA, *Drosophila melanogaster* mannosidase II, human GnTI, and rat GnTII were introduced into an *och1* knockout strain, resulting in the homogeneous formation of the complex human GlcNAc_2_Man_3_GlcNAc_2_ glycan [64]. In other studies, *OCH1* was inactivated via a knock-in strategy [65], an ER-targeted HEDL (His-Asp-Glu-Leu; C-terminal tetrapeptide involved in the lumen sorting of soluble proteins)-tagged α-1,2-mannosidase from *Trichoderma reesei* was introduced, and a chimeric human GnTI was fused to the N-terminal part of *Saccharomyces cerevisiae* Kre2 for Golgi localization [66]. A further approach included the construction of a strain expressing mouse mannosidase IA, the *K. lactis* UDP-GlcNAc transporter, human GnTI, and rat GnTII, in which the *ALG3* gene, encoding an α-1,3-mannosyltransferase of the ER lumen, was knocked out [67], leading to the formation of GlcNAc_2_Man_3_GlcNAc_2_. Additional coexpression of a fusion protein consisting of the *S. cerevisiae* Mnn2 Golgi localization domain and the activities of *Schizosaccharomyces pombe* UDP-Gal 4-epimerase and human β-1,4-galactosyl transferase allowed the production of Ga_l2_GlcNAc_2_Man_3_GlcNAc_2_ glycans. An alternative protocol allowed production of Gal_2_GlcNAc_2_Man_3_GlcNAc_2_ N-glycans using the GlycoSwitch vector technology [68], where specially designed vectors are used to replace genes of the native glycosylation pathway. Further humanization was achieved by additional biosynthesis of cytidine monophosphate-linked Sia, its transport and the transfer of Sia onto the N-glycans of nascent polypeptides, leading to complex human Sia_2_Gal_2_GlcNAc_2_Man_3_GlcNAc_2_ glycans [69]. Additional glycoengineering studies included the elimination of α-1,2-mannosidase-resistant high Man glycans [70] and overexpression of *Leishmania major* STT3D to increase N-glycan site occupancy [71]. These steps make it possible to use glycoengineered *P. pastoris* strains for the production of full length mAbs (e.g., [72]).

#### Production process

In contrast to *S. cerevisiae*, *P. pastoris* prefers respiratory over fermentative growth, allowing cultivations to very high cell densities, for example, 160 g/l cell dry weight [37], on inexpensive, defined media without the risk of accumulating ethanol. The very well-studied production processes in *P. pastoris* are most commonly performed as fed-batch processes. The possibility of performing mixed-feed fed-batch cultivations, where two substrates are concomitantly fed facilitating biomass growth due to higher biomass yields on the second substrate and leading to lower oxygen consumption and lower heat production, is a significant advantage of yeasts over mammalian cells and has already been applied successfully for the production of scFvs with *P. pastoris* [38]. In addition, a recent study presented a dynamic approach for determining strain-specific parameters in simple batch cultivations. This approach enables the design of efficient mixed-feed strategies for this yeast [39].

In conclusion, *P. pastoris* is a well-established host system for the production of antibody fragments. In fact, two recombinant therapeutic antibody fragments are already on the market: Nanobody ALX0061, which is a recombinant anti-IL6 receptor single domain antibody fragment used for rheumatoid arthritis treatment, and Nanobody^®^ ALX00171, a recombinant anti-RSV single domain antibody fragment used for respiratory syncytial virus (RSV) infection treatment. Given recent and ongoing advances in glycoengineering, *P. pastoris* is of increasing interest for the production of glycosylated full length mAbs (Box 2).

### The bacterium E. coli

Due to rapid growth on inexpensive substrates, the ability to reach high cell densities, well-understood genetics, and easy genetic manipulation, prokaryotic expression systems are widely used for the production of recombinant proteins. The gram-negative bacterium *E. coli* was the first microbial organism employed for the production of recombinant biopharmaceuticals and still accounts for nearly 40% of all the marketed biopharmaceutical compounds produced today. After the approval of humulin in 1982, several different therapeutic proteins, such as antibody fragments [e.g., the antitumor necrosis factor (TNF)-α Fab], have been successfully produced in this prokaryotic organism [11].

#### Expression system

Recombinant protein expression in bacterial hosts is generally driven by self-replicating multicopy plasmids carrying a strong promoter, like the bacteriophage T7, the *E. coli* lactose operon *(lac)* or the synthetic tryptophan operon (*trp*) promoter, and a ribosome binding site allowing high gene dosages [40]. Although greater volumetric productivity can be reached by implementing self-replicating multicopy plasmids, these cause a severe metabolic burden for *E. coli*, including cell growth inhibition and cell death. Thus, new plasmid-free expression systems, based on site-directed chromosomal integration of the heterologous DNA, have been developed [41]. In order to eliminate the metabolic burden associated with the selection marker, a novel marker-free plasmid selection system using a genomically modified *E. coli* strain was also engineered [42].

#### Strain engineering

Although cytoplasmic production in *E. coli* allows high intracellular product yields, it is often associated with inclusion body formation (e.g., [43]). This *E. coli* characteristic phenomenon arises from unbalanced expression of folding helper elements and the fact that disulfide bridges cannot be formed correctly in the reductive environment of the cytoplasm. This problem can be overcome by the co-expression of chaperones [44] or by the transport of the target protein to the periplasmic space by fusion to a leader peptide at the N terminus [45]. Secretion into the periplasm has already been successfully performed for antibody fragments [46]. However, if efficient refolding is possible, recombinant protein production in inclusion bodies also describes a valuable production strategy, as already described for Fc-fusion proteins [47].

#### Production process

Due to the intrinsic high growth rate of *E. coli*, high cell density cultures are currently used for the production of antibody fragments [48]. Production processes with *E. coli* are commonly conducted in stirred tank reactors (STR) as limited glucose fed-batch processes because glucose excess induces overflow metabolism and causes the production of the inhibiting metabolite acetate. As an alternative to a carbon source-limited feeding strategy, different metabolic engineering approaches have been designed to prevent or at least reduce acetate formation. These approaches include manipulating the native acetate formation pathway [49] and engineering the endogenous glucose uptake system [50]. Another recent advance is to improve the bioprocess via the identification and characterization of key strain-specific physiological parameters instead of excessive strain engineering. The knowledge of the strain characteristic parameters specific substrate uptake rate (q_s_) and maximum specific substrate uptake rate (q_s__max_), for example, allows the design of tailored bioprocesses avoiding overflow metabolism. Soft-sensor tools, which are virtual sensors processing different signals measured online that give real-time information on a non-measurable process parameter, are powerful tools for that purpose [51]. Besides, strain-specific physiological parameters can easily be determined by applying dynamic changes of process parameters during cultivation [52]. The availability of detailed physiological data enables design that follows the Quality by Design (QbD) guidelines [53].

In summary, antibody fragments, which are not glycosylated, can be produced in *E. coli* and the required tools are already in place (e.g., [11]). Remarkably, successful production of full length mAbs in *E. coli* was achieved recently, although the mAbs were not glycosylated [47]. The identification of the N-glycosylation pathway in *Campylobacter jejuni* and the possibility of introducing it into *E. coli* [54] may pave the way for the production of the glycosylated Fc domain [55] and the successful expression of full length mAbs in *E. coli*. For this reason, pharmaceutical companies are now investing effort and capital in re-introducing *E. coli* to their production facilities.

## Concluding remarks and future perspectives

Full length mAbs as well as antibody fragments represent the most important and valuable class of biopharmaceuticals today. Due to the requirement for surface glycosylation, mAbs are still predominantly produced in mammalian cells, which possess several drawbacks relating to bioprocessing and scale-up. By contrast, antibody fragments, which are not glycosylated but retain antigen binding properties, can also be produced in microbial organisms. Recent advances in the production of full length mAbs and antibody fragments with both mammalian cells and microbials are summarized in Table 1.

**Table 1:** Recent advances in the production of full length mAbs and antibody fragments with different host organisms

Production milestone	Recent advances					
	Mammalian cells	Refs	Yeasts	Refs	*Escherichia coli*	Refs
Stable and efficient expression system	Site-specific homologous recombination Vector engineering and marker attenuation Expression of anti-apoptotic genes	[73] 74, 75 [76]	Targeted gene integration Concomitant expression of several genes Co-expression of chaperones Reduction of proteolysis Over-expression of Hsf	[15] [17] [34] 21, 35 [22]	Plasmid-free expression system Marker-free selection system Co-expression of chaperones	[41] [42] [44]
Clone selection	Robotics and fluorescence-activated cell sorting	[75]	Targeted gene integration Optimization of codons, gene copy number, and promoters	[15] [77]	Not an issue	
Disulfide bridges	Intrinsic feature of the ER		Intrinsic feature of the ER		Transport to the periplasm	[46]
Product secretion	Intrinsic feature		Over-expression of SNAREs Mutation studies on MFα1 System biological analysis	[20] [78] [77]	Transport to the periplasm	[46]
Chemically defined medium (CFD)	Serum-free CFD	[56]	Already applied		Already applied	
Efficient bioprocess	Concentrated fed-batch strategy	[79]	Fully aerobic strain Dynamic processes	[23] [80]	Manipulating the native acetate formation pathway Engineering the glucose uptake system	[49] [50]

As shown in Table 1, current efforts are directed towards optimizing the production of mAbs and antibody fragments in microbial organisms, because they outpace mammalian cells in several aspects, such as the ease of genetic manipulation, greater productivity, and high cell density cultivation processes on inexpensive and defined substrates. Although mAbs are still most frequently produced in mammalian cells, ongoing glycoengineering studies with yeasts (Box 2) and *E. coli* 54, 55 are paving the way for the successful production of glycosylated full length mAbs in microbial host organisms.

## References

[1] Lee J.Y., Bang D. (2010). Challenges in the chemical synthesis of average sized proteins: sequential vs. convergent ligation of multiple peptide fragments. Biopolymers.

[2] Bolivar J.M. (2013). Shine a light on immobilized enzymes: real-time sensing in solid supported biocatalysts. Trends Biotechnol..

[3] Chelliapan S., Sallis P.J. (2013). Removal of organic compound from pharmaceutical wastewater using advanced oxidation processes. J. Sci. Ind. Res..

[4] Walsh G. (2012). Process Development Forum.

[5] Butler M., Meneses-Acosta A. (2012). Recent advances in technology supporting biopharmaceutical production from mammalian cells. Appl. Microbiol. Biotechnol..

[6] Berlec A., Strukelj B. (2013). Current state and recent advances in biopharmaceutical production in Escherichia coli, yeasts and mammalian cells. J. Ind. Microbiol. Biotechnol..

[7] de Marco A. (2011). Biotechnological applications of recombinant single-domain antibody fragments. Microb. Cell Fact..

[8] Li F. (2010). Cell culture processes for monoclonal antibody production. MAbs.

[9] Ahmad Z.A. (2012). scFv antibody: principles and clinical application. Clin. Dev. Immunol..

[10] Nelson A.L. (2010). Antibody fragments: hope and hype. MAbs.

[11] Walsh G. (2010). Biopharmaceutical benchmarks 2010. Nat. Biotechnol..

[12] Gorlani A. (2012). Expression of VHHs in *Saccharomyces cerevisiae*. Methods Mol. Biol..

[13] Chee M.K., Haase S.B. (2012). New and redesigned pRS plasmid shuttle vectors for genetic manipulation of *Saccharomyces cerevisiae*. G3 (Bethesda).

[14] Park Y.N. (2011). Application of the FLP/FRT system for conditional gene deletion in yeast *Saccharomyces cerevisiae*. Yeast.

[15] Leite F.C. (2013). Construction of integrative plasmids suitable for genetic modification of industrial strains of *Saccharomyces cerevisiae*. Plasmid.

[16] Partow S. (2010). Characterization of different promoters for designing a new expression vector in *Saccharomyces cerevisiae*. Yeast.

[17] Maury J. (2008). Reconstruction of a bacterial isoprenoid biosynthetic pathway in *Saccharomyces cerevisiae*. FEBS Lett..

[18] Joosten V. (2003). The production of antibody fragments and antibody fusion proteins by yeasts and filamentous fungi. Microb. Cell Fact..

[19] Xu P. (2005). Analysis of unfolded protein response during single-chain antibody expression in *Saccharomyces cerevisiae* reveals different roles for BiP and PDI in folding. Metab. Eng..

[20] Hou J. (2012). Engineering of vesicle trafficking improves heterologous protein secretion in *Saccharomyces cerevisiae*. Metab. Eng..

[21] Idiris A. (2010). Enhanced protein secretion from multiprotease-deficient fission yeast by modification of its vacuolar protein sorting pathway. Appl. Microbiol. Biotechnol..

[22] Hou J. (2013). Heat shock response improves heterologous protein secretion in *Saccharomyces cerevisiae*. Appl. Microbiol. Biotechnol..

[23] Ferndahl C. (2010). Increasing cell biomass in *Saccharomyces cerevisiae* increases recombinant protein yield: the use of a respiratory strain as a microbial cell factory. Microb. Cell Fact..

[24] Hamilton S.R., Gerngross T.U. (2007). Glycosylation engineering in yeast: the advent of fully humanized yeast. Curr. Opin. Biotechnol..

[25] Chiba Y. (1998). Production of human compatible high mannose-type (Man5GlcNAc2) sugar chains in *Saccharomyces cerevisiae*. J. Biol. Chem..

[26] Mattia, A. Diversa Corporation (2006) GRAS notification concerning BD16449 – phospholipase C enzyme preparation from *Pichia pastoris*, http://www.accessdata.fda.gov/scripts/fcn/gras_notices/grn000204.pdf

[27] Naatsaari L. (2012). Deletion of the *Pichia pastoris* KU70 homologue facilitates platform strain generation for gene expression and synthetic biology. PLoS ONE.

[28] Krainer F.W. (2012). Recombinant protein expression in *Pichia pastoris* strains with an engineered methanol utilization pathway. Microb. Cell Fact..

[29] Delic M. (2013). Repressible promoters – a novel tool to generate conditional mutants in *Pichia pastoris*. Microb. Cell Fact..

[30] Ruth C. (2010). Variable production windows for porcine trypsinogen employing synthetic inducible promoter variants in *Pichia pastoris*. Syst. Synth. Biol..

[31] De Schutter K. (2009). Genome sequence of the recombinant protein production host *Pichia pastoris*. Nat. Biotechnol..

[32] Mattanovich D. (2009). Open access to sequence: browsing the *Pichia pastoris* genome. Microb. Cell Fact..

[33] Sohn S.B. (2010). Genome-scale metabolic model of methylotrophic yeast *Pichia pastoris* and its use for *in silico* analysis of heterologous protein production. Biotechnol. J..

[34] Inan M. (2006). Enhancement of protein secretion in *Pichia pastoris* by overexpression of protein disulfide isomerase. Biotechnol. Bioeng..

[35] Boehm T. (1999). Disruption of the KEX1 gene in *Pichia pastoris* allows expression of full-length murine and human endostatin. Yeast.

[36] Baumann K. (2011). Protein trafficking, ergosterol biosynthesis and membrane physics impact recombinant protein secretion in *Pichia pastoris*. Microb. Cell Fact..

[37] Jahic M. (2002). Modeling of growth and energy metabolism of *Pichia pastoris* producing a fusion protein. Bioprocess Biosyst. Eng..

[38] Hellwig S. (2001). Analysis of single-chain antibody production in *Pichia pastoris* using on-line methanol control in fed-batch and mixed-feed fermentations. Biotechnol. Bioeng..

[39] Zalai D. (2012). A dynamic fed batch strategy for a *Pichia pastoris* mixed feed system to increase process understanding. Biotechnol. Prog..

[40] Tegel H. (2011). Enhancing the protein production levels in *Escherichia coli* with a strong promoter. FEBS J..

[41] Striedner G. (2010). Plasmid-free T7-based *Escherichia coli* expression systems. Biotechnol. Bioeng..

[42] Mairhofer J. (2010). Marker-free plasmids for gene therapeutic applications –Lack of antibiotic resistance gene substantially improves the manufacturing process. J. Biotechnol..

[43] Khodabakhsh F. (2013). Comparison of the cytoplasmic and periplasmic production of reteplase in *Escherichia coli*. Prep. Biochem. Biotechnol..

[44] Sonoda H. (2011). Effects of cytoplasmic and periplasmic chaperones on secretory production of single-chain Fv antibody in *Escherichia coli*. J. Biosci. Bioeng..

[45] Yuan J.J. (2010). Protein transport across and into cell membranes in bacteria and archaea. Cell. Mol. Life Sci..

[46] Levy R. (2013). Enhancement of antibody fragment secretion into the *Escherichia coli* periplasm by co-expression with the peptidyl prolyl isomerase, FkpA, in the cytoplasm. J. Immunol. Methods.

[47] Huang C.J. (2012). Industrial production of recombinant therapeutics in *Escherichia coli* and its recent advancements. J. Ind. Microbiol. Biotechnol..

[48] Jalalirad R. (2013). Production of antibody fragment (Fab) throughout *Escherichia coli* fed-batch fermentation process: changes in titre, location and form of product. Electron. J. Biotechnol..

[49] Tao Y. (2012). Metabolic engineering for acetate control in large scale fermentation. Methods Mol. Biol..

[50] Lara A.R. (2008). Utility of an *Escherichia coli* strain engineered in the substrate uptake system for improved culture performance at high glucose and cell concentrations: an alternative to fed-batch cultures. Biotechnol. Bioeng..

[51] Sagmeister P. (2013). Soft sensor assisted dynamic bioprocess control: efficient tools for bioprocess development. Chem. Eng. Sci..

[52] Jazini M., Herwig C. (2011). Effect of post-induction substrate oscillation on recombinant alkaline phosphatase production expressed in *Escherichia coli*. J. Biosci. Bioeng..

[53] Wechselberger P. (2013). Model-based analysis on the extractability of information from data in dynamic fed-batch experiments. Biotechnol. Prog..

[54] Fisher A.C. (2011). Production of secretory and extracellular N-linked glycoproteins in *Escherichia coli*. Appl. Environ. Microbiol..

[55] Lizak C. (2011). N-Linked glycosylation of antibody fragments in *Escherichia coli*. Bioconjug. Chem..

[56] van der Valk J. (2010). Optimization of chemically defined cell culture media–replacing fetal bovine serum in mammalian in vitro methods. Toxicol. In Vitro.

[57] Kim J.Y. (2011). Proteomic understanding of intracellular responses of recombinant Chinese hamster ovary cells cultivated in serum-free medium supplemented with hydrolysates. Appl. Microbiol. Biotechnol..

[58] Ning D. (2005). Production of recombinant humanized anti-HBsAg Fab fragment from *Pichia pastoris* by fermentation. J. Biochem. Mol. Biol..

[59] Wildt S., Gerngross T.U. (2005). The humanization of N-glycosylation pathways in yeast. Nat. Rev. Microbiol..

[60] Mille C. (2008). Identification of a new family of genes involved in beta-1,2-mannosylation of glycans in *Pichia pastoris* and *Candida albicans*. J. Biol. Chem..

[61] Cregg J.M. (1993). Recent advances in the expression of foreign genes in *Pichia pastoris*. Biotechnology.

[62] Choi B.K. (2003). Use of combinatorial genetic libraries to humanize N-linked glycosylation in the yeast *Pichia pastoris*. Proc. Natl. Acad. Sci. U.S.A..

[63] Nett J.H. (2011). A combinatorial genetic library approach to target heterologous glycosylation enzymes to the endoplasmic reticulum or the Golgi apparatus of *Pichia pastoris*. Yeast.

[64] Hamilton S.R. (2003). Production of complex human glycoproteins in yeast. Science.

[65] Bernett M.J. (2010). Engineering fully human monoclonal antibodies from murine variable regions. J. Mol. Biol..

[66] Callewaert N. (2001). Use of HDEL-tagged *Trichoderma reesei* mannosyl oligosaccharide 1,2-alpha-D-mannosidase for N-glycan engineering in *Pichia pastoris*. FEBS Lett..

[67] Davidson R.C. (2004). Functional analysis of the ALG_3_ gene encoding the Dol-P-Man: Man_5_GlcNAc_2_-PP-Dol mannosyltransferase enzyme of *P. pastoris*. Glycobiology.

[68] Jacobs P.P. (2009). Engineering complex-type N-glycosylation in *Pichia pastoris* using GlycoSwitch technology. Nat. Protoc..

[69] Hamilton S.R. (2006). Humanization of yeast to produce complex terminally sialylated glycoproteins. Science.

[70] Hopkins D. (2011). Elimination of beta-mannose glycan structures in *Pichia pastoris*. Glycobiology.

[71] Choi B.K. (2012). Improvement of N-glycan site occupancy of therapeutic glycoproteins produced in *Pichia pastoris*. Appl. Microbiol. Biotechnol..

[72] Ye J. (2011). Optimization of a glycoengineered *Pichia pastoris* cultivation process for commercial antibody production. Biotechnol. Prog..

[73] Campbell M. (2010). Utilization of site-specific recombination for generating therapeutic protein producing cell lines. Mol. Biotechnol..

[74] Kameyama Y. (2010). An accumulative site-specific gene integration system using Cre recombinase-mediated cassette exchange. Biotechnol. Bioeng..

[75] Lai T. (2013). Advances in mammalian cell line development technologies for recombinant protein production. Pharmaceuticals.

[76] Becker E. (2010). Evaluation of a combinatorial cell engineering approach to overcome apoptotic effects in XBP-1(s) expressing cells. J. Biotechnol..

[77] Pfeffer M. (2012). Intracellular interactome of secreted antibody Fab fragment in *Pichia pastoris* reveals its routes of secretion and degradation. Appl. Microbiol. Biotechnol..

[78] Lin-Cereghino G.P. (2013). The effect of alpha-mating factor secretion signal mutations on recombinant protein expression in *Pichia pastoris*. Gene.

[79] Lim S. (2011). An economic comparison of three cell culture techniques. BioPharm. Int..

[80] Dietzsch C. (2011). A fast approach to determine a fed batch feeding profile for recombinant *Pichia pastoris* strains. Microb. Cell Fact..

